# PALLIATIVE CARE KNOWLEDGE, HEALTH CONCERNS, AND EDUCATIONAL STATUS IN DECLINING COMMUNITY-BASED PALLIATIVE CARE

**DOI:** 10.1093/geroni/igae098.2061

**Published:** 2024-12-31

**Authors:** Elizabeth Luth, Abigail Amponsah

**Affiliations:** Rutgers University, New Brunswick, New Jersey, United States; Newark Board of Education, Newark, New Jersey, United States

## Abstract

Palliative care in community settings can support seriously ill individuals to reduce symptoms and maintain quality of life. However, many individuals decline palliative care, even when they are concerned about their health. Differences in understanding how palliative care can help manage serious illness may contribute to individuals declining. This study explores the relationship between individuals’ understanding of palliative care, their health concerns, and education levels (a proxy for health literacy). We use survey responses from a validated instrument assessing palliative care knowledge and information from semi-structured interviews for 21 seriously ill individuals (n=3) and family caregivers (n=18) who were eligible for community-based palliative care services and declined. We explore the relationship between respondents’ education, understanding of palliative care, and their concerns about their or their loved one’s health. Among participants, twelve had a bachelor’s degree or higher; nine had some college or less. A higher percentage of participants with some college or less were concerned about psychological issues, stress, and managing medication side effects (67% vs 42%). All participants who did not understand palliative care could help with stress and medication side effects and were concerned about these had some college or less. One of three participants who did not understand palliative care could help with psychological issues and were concerned about these had some college or less. Assessing health concerns and explaining how palliative care can address those concerns may reduce barriers to accepting palliative care, particularly among individuals with lower levels of education.

